# A New Method of Deep Convolutional Neural Network Image Classification Based on Knowledge Transfer in Small Label Sample Environment

**DOI:** 10.3390/s22030898

**Published:** 2022-01-25

**Authors:** Yunchen Kong, Xue Ma, Chenglin Wen

**Affiliations:** 1 School of Automation, Hangzhou Dianzi University, Hangzhou 310018, China; kongyc17855305699@163.com (Y.K.); xuema@hdu.edu.cn (X.M.); 2 School of Automation, Guangdong University of Petrochemical Technology, Maoming 525000, China

**Keywords:** support vector machine, convolutional neural network, knowledge transfer, bag of visual words

## Abstract

The problem of deep learning network image classification when a large number of image samples are obtained in life and with only a small amount of knowledge annotation, is preliminarily solved in this paper. First, a support vector machine expert labeling system is constructed by using a bag-of-words model to extract image features from a small number of labeled samples. The labels of a large number of unlabeled image samples are automatically annotated by using the constructed SVM expert labeling system. Second, a small number of labeled samples and automatically labeled image samples are combined to form an augmented training set. A deep convolutional neural network model is created by using an augmented training set. Knowledge transfer from SVMs trained with a small number of image samples annotated by artificial knowledge to deep neural network classifiers is implemented in this paper. The problem of overfitting in neural network training with small samples is solved. Finally, the public dataset caltech256 is used for experimental verification and mechanism analysis of the performance of the new method.

## 1. Introduction

Today’s world is in an era of big data. Social networking sites, intelligent transportation, intelligent medicines, and other fields are generating massive image samples every day. These massive data provide the premise and support for the training and updating of deep learning models [1,2,3]. The serious problem of big data is that the number of samples is large, but the labeled samples that endow knowledge are few. This problem hinders the generalization of deep convolutional neural network models with strong learning and generalization capabilities in real life [4]. In order to solve this problem, the first task is how to expand the existing small number of labeled samples to generate a large number of labeled image samples that meet the needs of deep learning model training [5,6]. Secondly, due to the high dimension of the image, the existing expert systems that only classify low-dimensional image features well, are difficult to apply to the expansion of image annotation samples. The key to solving the above problems is to achieve effective feature dimensionality reduction for existing high-dimensional images. The final problem to be solved to achieve the goal is the selection of a suitable deep learning image classification model and model parameter training method [7,8,9,10,11]. When the number of network layers is deep, Kalman filtering can be used to adaptively update the neural network [12,13].

The authors in [14] studied the effects of unbalanced data sets on different classifiers. This document uses a data balance algorithm to effectively improve the classification performance of the classifier in the unbalanced data. This study details the effects of sampling inadequate data sets, using balance algorithms and over-sampling data sets on classifier performance. It has proven to have an important role in the performance of a few types of data sets. The authors in [15] proposed a new loss function in the model training phase, the squared gradient magnitude loss (SGM) was used to improve the effect of representation learning. The literature used generators to generate new data for data augmentation for few-shot classes. The disadvantage of this method is that it captures complex data distributions. The authors in [16] combined meta-learning and data augmentation to generate new samples with different variations by changing illumination, location, etc. The authors in [17] proposed a prototype network. The prototype network used a deep neural network to map images into vectors. For samples belonging to the same category, the average value of this category of sample vectors was obtained as the prototype of the category. By continuously training the model and minimizing the loss function, the distance between samples in the same category were closer together, and the distance between samples in different categories were further away, so as to update the parameters of the embedding function. The idea and implementation method of the prototype network are very simple, but the results obtained by only using the labeled data are not necessarily accurate, and the sample size is too small, which will lead to the deviation of the classification boundary. The authors in [18] proposed a method for solving the few-shot problem with federated learning. The authors in [19] proposed a neural network training method from the source domain to the target domain. Mehrotra et al. used the generative adversarial network to solve the small sample problem. First, input the sample data and destroy it, then input the damaged sample into the generator network to generate a new sample, and compare the new sample with the original input sample to judge whether the new sample is a real sample. The algorithm effectively expands the experimental data by synthesizing new samples [20]. However, when it is in the deep learning framework, it needs a certain number of training samples to generate the countermeasure network. Boltzmann machine generates a probability model from samples. The model parameters are infinite dimensions. A considerable number of samples are required to generate the function. Compared with generating a countermeasure network and Boltzmann machine, this paper realizes the effective training of a neural network based on only labeled small samples, and it only needs a small number of training samples. The reason is that there are relatively few SVM parameters. Based on a small number of labeled samples, a mature SVM classification model can be trained to assign knowledge to unlabeled image samples, so as to realize the capacity expansion training of the neural network.

Our core contribution is to creatively train a deep neural network image classifier with a large number of parameters using small labeled samples. The executable bridge is built between a small number of labeled samples and the generation of a large number of samples that meet the needs of neural networks. The specific steps are as follows. First, based on a small number of image sample sets annotated by artificial knowledge, three feature descriptors are used to extract the underlying features of the image. The visual word bag feature vector set is constructed by clustering. The clustering method achieves effective dimensionality reduction for complex features in original images. Secondly, the support vector machine image classification model was constructed, respectively based on the established visual word bag feature vector set. Thirdly, a large number of image sample sets without knowledge annotation obtained in life was input into the established support vector machine image classification model in turn, and the labels of the corresponding samples were predicted. Then, the selected deep convolutional neural network classification model parameters were trained based on the obtained set of a large number of annotated image samples. Finally, the classification performance of the neural network after knowledge transfer was verified by experiments. The experimental results show that under the different number of predicted labels, with the continuous increase in the number of enhanced training sets of the neural network, its classification accuracy continued to improve. Our method is 3% higher than the highest average classification accuracy of H0G_BOVW_SVM. Compared with the convolutional neural network with parameter initialization under the few-label samples, the average classification accuracy of our method is 20% higher. The expert knowledge reflected by the designed shallow support vector machine model is successfully transferred to the convolutional neural network in the form of predicted labels, which improves the shortcomings of the convolutional neural network’s poor expressive ability and generalization ability due to insufficient labeled samples.

## 2. Visual Word Bag Feature Extraction and SVM Image Classifier Construction Based on Small Label Image Samples

The main purpose of this section is to construct a shallow support vector machine model based on a small number of manually labeled samples for the data expression of expert knowledge: Firstly, three different feature descriptors are used to extract the underlying features of the image [21,22], and the visual word bag model is created by clustering method to realize the effective dimensionality reduction in complex features. The constructed visual word bag model is used to represent the image features [23,24,25,26]. Then, based on the established visual word bag feature vector set, support vector machine image classification models with a small number of knowledge label samples as input are constructed, respectively. Finally, a large number of unlabeled image samples are input into the established support vector machine with good classification performance to assign knowledge annotation.

### 2.1. Construction of the Bag of Visual Word Based on Small Label Image Samples

The main objective of this section is to extract the bottom features of the sample image based on the image sample set marked with a small amount of artificial knowledge in real life, and then generate the visual word bag feature vector set by the K-means clustering method, so as to effectively reduce the dimension of complex features in the marked sample image; it lays a foundation for establishing the SVM image classification method based on low dimension.

#### 2.1.1. Image Bottom Feature Extraction Based on Three Feature Descriptors

The BOVW model is widely used in traditional image classification algorithms. Its main idea is to map the local low-level features to the middle and high-level latent semantic features of the image through clustering algorithm. Each cluster center is the visual word of the image, and all cluster centers form a visual word bag, so that each image can be represented by the visual word frequency histogram. Because the quality of the visual word bag is closely related to the extracted bottom features, and the quality of the visual word bag will further affect the accuracy of image classification, three different feature descriptors are selected to extract the bottom features of image, which are scale invariant feature transform, histogram of oriented gradient, and Canny edge detection algorithm.

Sift describes the image by finding local interest points in different scale spaces to help identify objects. SIFT divides the image into 4 × 4 grid. In each grid, the gradient is quantified into 8 directions to form a 128-dimensional vector. Hog first converts the image into a gray image, and then calculates the gradient value of each pixel to capture texture information and contour. Canny realizes edge detection by using non maximum and double threshold detection. Its advantage is that it can detect weak edge details.

#### 2.1.2. Bag of Visual Word Were Generated Based on Image Underlying Features and the K-Means Clustering Method

**Figure 1:**
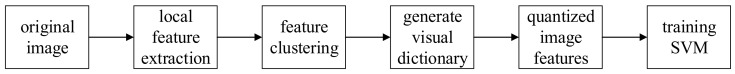
Flow chart of Bag of Visual Words.

Based on the features extracted by the bottom feature descriptor, the effective dimensionality reduction in the features is realized by the clustering method, and the visual word bag of the image is further generated. The algorithm flow chart of bag of the visual word model is shown in Figure 1 below:

The image classification algorithm based on bag of visual word model is divided into four steps:

The first step is to extract the local feature vector of the image with the above three different descriptors.

In the second step, due to the large number of feature vectors extracted by the feature descriptor and high dimension, the K-means clustering method is used to cluster the bottom local feature vectors of the image obtained by the feature descriptor into K clusters, so that there is high similarity in the clusters, but low similarity between clusters, and each cluster center is a visual word.

In the third step, the image is represented as the distribution of words, and the visual word frequency histogram is used to represent the image features. After K-means clustering, a visual dictionary composed of K visual words is obtained, which can be expressed as $V=(v_{1},v_{2},\cdots v_{i},\cdots ,v_{k})$, $v_{i}$ represents a visual word. In this way, the image sample is represented as a k-dimensional feature vector F, in the specific form of $F=(f_{1},f_{2},\cdots f_{i},\cdots ,f_{k})$, where $f_{i}$ is visual word $v_{i}$ number of occurrences.

In the fourth step, the classifier support vector machine is designed and trained, and the image feature vector represented by word bag model is used for image classification.

### 2.2. Construction of the SVM Classifier Based on Bag of Visual Word of Small Label Image Samples

The main goal of this section is to build a support vector machine image classification model with a small number of labeled samples as input based on the established visual word bag feature vector set. A large number of unlabeled image samples are input into the established support vector machine with good classification performance for label assignment.

**Figure 2:**
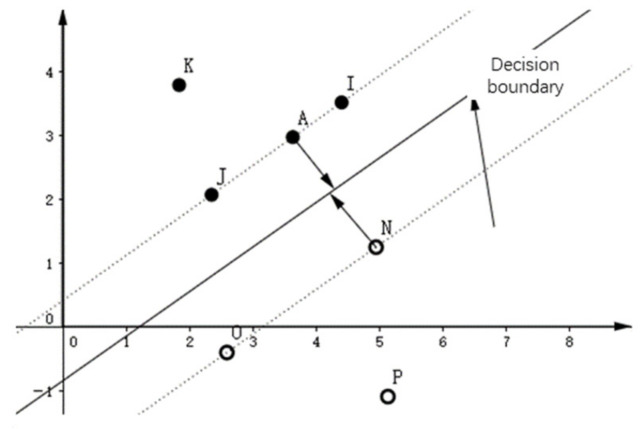
Schematic diagram of support vector machine.

SVM is a new structured learning method based on structural risk minimization. SVM shows excellent performance in the case of small samples and the creation of the model is not complex. When the kernel function is introduced, it can solve various kinds of nonlinear problems. Support vector machine classifier is a classifier that aims to find the maximum margin to solve the parameters w and b, so as to construct the decision boundary and classify with the decision boundary. The schematic diagram of SVM is shown in Figure 2.

For non-linear separable sample data, it is necessary to increase the dimension of the data and project the data from the original space x to the new space Φ(x). The decision function of SVM dealing with nonlinear problems is as follows:

(1) $$f(x_{test})=sign(w\cdot \Phi (x_{test})+b)=sign(\sum_{i=1}^{N}\alpha_{i}y_{i}\Phi (x_{i})\cdot \Phi (x_{test})+b)$$

Among them, $\alpha_{i}$ is the Lagrange multiplier. $\left(x_{i},y_{i}\right)$ is the training set sample input by the support vector machine. N is the number of training set samples input by the support vector machine, and $x_{test}$ is the test set sample of the support vector machine.

In extreme cases, the kernel trick is used by SVMs to solve multi-dimensional and computationally expensive problems because the data may be mapped into an infinite-dimensional space. The kernel trick is a mathematical way of representing the result of a dot product in the increased dimensional space using a vector calculation in the original space of the data. Specifically:

(2) K(u,v)=Φ(u)⋅Φ(v)

The dot product function K(u,v) in the original space is called kernel function. Φ(x) is a mapping function. With the kernel function, the decision boundary is expressed as:

(3) $$f(x_{test})=sign(\sum_{i=1}^{N}\alpha_{i}y_{i}\Phi (x_{i})\cdot \Phi (x_{test})+b)=sign(\sum_{i=1}^{N}\alpha_{i}y_{i}K(x_{i},x_{test})+b)$$

The kernel function is calculated in the original space, which can avoid the problem of the curse of dimensionality. In this paper, the linear kernel function is selected for image classification test.

**Figure 3:**
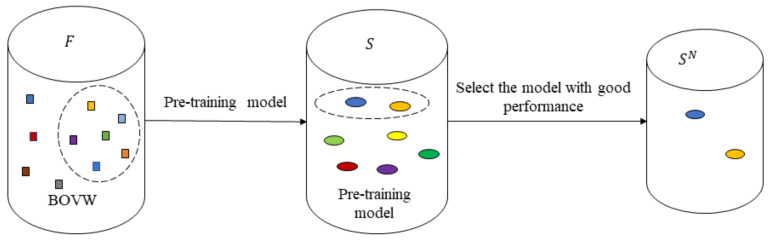
Flow chart of pre training model.

In Section 2.1, different feature extraction methods are used to extract the bottom features of the image, and the extracted features are clustered into word bags. Based on the different image features represented by different word bags, the classification accuracy of the trained shallow model support vector machine is also different. The model with the highest predicted label accuracy is selected as the expert system. The expert system labels and filters unlabeled image samples to form a quasi-labeled dataset. The specific process is shown in Figure 3. Multiple SVM expert systems are trained based on different image features of a small number of labeled samples. The mathematical expression is as follows:

(4) $$S=\{s_{i}\left(F_{i}\right)|F_{i}\subseteq F\}$$

where $s_{i}\left(\cdot \right)$ represents the ith expert system trained based on feature $F_{i}$.

Then, Rank is determined by the classification accuracy of $s_{i}$ and the first m expert systems with better classification performance are selected to construct a model candidate pool:

(5) $$S^{N}=\{s_{i}|Rank(Acc(s_{i}))\in \left\{0,1,\ldots ,m-1\right\}\}$$

Among them, m is taken as 2 in this paper, $Acc(s_{i})$ represents the classification accuracy of the model $s_{i}$ on the validation set, and its mathematical expression is

(6) $$Acc=\sum_{i=1}^{c}x_{ii}/\sum_{i=1}^{c}\sum_{j=1}^{c}x_{ij}$$

Among them, $x_{ij}$ is the number of samples that actually belong to the ith class, but is predicted by the model to be the jth class; c=6, refers to the number of categories to be distinguished. The pre-trained SVM model in $S^{N}$ is selected to predict unlabeled samples by measuring the accuracy rate.

(7) $$D^{u}=\left\{\left({\tilde{x}}_{l+1},{\tilde{y}}_{l+1}),({\tilde{x}}_{l+2},{\tilde{y}}_{l+2}),\cdots ,({\tilde{x}}_{l+u},{\tilde{y}}_{l+u}\right)\right\}$$

where ${\tilde{y}}_{l+u}$ is the prediction label of unlabeled image sample ${\tilde{x}}_{l+u}$. ${\tilde{y}}_{l+u}$ is obtained by fusing multiple SVM models:

(8) $${\tilde{y}}_{l+u}=g(s_{i}(F_{l+u})|s_{i}\in S^{N})$$

where g(⋅) represents the fusion function. The specific process is to calculate the arithmetic average value of image classification prediction probability of different models, and select the category with the highest probability as the fusion function result.

## 3. Knowledge Transfer and Depth CNN Image Classifier Model Based on Expert Annotation System

After the shallow support vector machine model is established, a large number of unlabeled images are successively input into the support vector machine for assignment, labeling, and screening. A large number of labeled samples marked by the expert system and the original sparse labels are combined into an enhanced training set. The augmented training set is used to train and solve the selected deep convolutional neural network classification model. This process realizes knowledge transfer from sparse labeled sample sets to deep neural network classifiers.

### 3.1. Knowledge Transfer of Fenerating a Large Number of Label Samples

**Figure 4:**
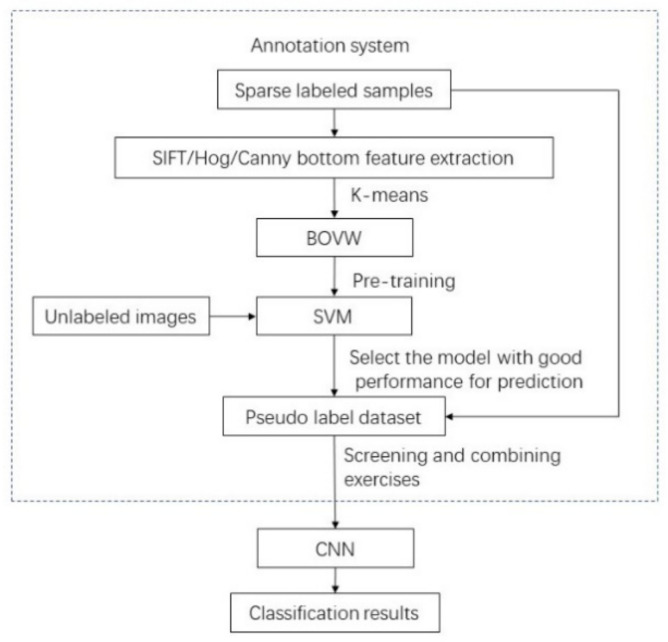
BOVW_SVM_ VGG16 algorithm flow chart.

The flow chart of BOVW_ SVM_VGG16 algorithm is shown in Figure 4. If the neural network is directly trained with sparse labeled samples, the neural network often suffers from severe overfitting. Therefore, this paper proposes a knowledge transfer-based convolutional neural network image classification method. Since different feature descriptors have different emphasis on image feature description, combining the advantages of three feature descriptors, the bag-of-words model is used to characterize image features, which are used to train a shallow model support vector machine. The SVM with the best training accuracy was used to make predictions on unlabeled images to form a quasi-labeled dataset. The predicted labels of quasi-label data sets are the data representation of SVM knowledge. In order to better extract the underlying features, this paper adopts three different feature extraction methods to build support vector machines. If you want to use other feature extraction methods, you can use the transfer learning method to transfer the feature extraction part of the mature neural network for image feature extraction, which is the future direction of work. After the support vector machine predicts the unlabeled samples, some of the labeled image samples and the predicted labeled samples are combined to form an enhanced training set, and the remaining part of the labeled samples is used as the test set. The augmented training set is randomly cropped, and the obtained image sample size is 224 × 224, and normalized preprocessing is performed. The image samples preprocessed by the data are sent to the selected neural network for training, where the loss function is the cross-entropy loss function, and the learning rate is 0.0001.

This study was conducted under the following assumptions:

The mathematical expression of labeled image sample data set is as follows:

(9) $$X_{l}=\left\{\left(x_{1},y_{1}),(x_{2},y_{2}),\cdots ,(x_{l},y_{l}\right)\right\}$$

where $x_{l}$ is the lth image sample, $y_{l}\in \left\{1,2,\cdots ,c\right\}$ is the class label of $x_{l}$, there are c classes in total. The mathematical expression of unlabeled image sample set is $X_{u}=\left\{x_{l+1},x_{l+2},\cdots ,x_{l+u}\right\}$.

Step: 1. Train SVM by minimizing the objective function.

Input: Part of the data is extracted from the labeled image sample set as the training set, the training set image data is $D_{T}^{S}=\left\{\left(x_{1},y_{1}),(x_{2},y_{2}),\cdots (x_{N},y_{N}\right)\right\}$, where i=1,2,⋯,N.

(1) The optimization problem is constructed and solved by selecting the appropriate kernel function and the appropriate parameter C.
  (10) $$\left\{\begin{array}{l} \underset{\alpha }{\min}\frac{1}{2}\sum_{i=1}^{N}\sum_{j=1}^{N}\alpha_{i}\alpha_{j}y_{i}y_{j}K(x_{i},y_{j})-\sum_{i=1}^{N}\alpha_{i}\\ s.t.\sum_{i=1}^{N}\alpha_{i}y_{i}=0\\ 0\leq \alpha_{i}\leq C,i=1,2,\cdots ,N \end{array}\right.$$
  Find the optimal solution $\alpha^{*}={\left(\alpha_{1}^{*},\alpha_{2}^{*},\cdots ,\alpha_{N}^{*}\right)}^{T}$.
(2) Choose $\alpha^{*}$ positive component of $0<\alpha_{j}^{*}<C$, calculation
  (11) $$b^{*}=y_{j}-\sum_{i=1}^{N}\alpha_{i}^{*}y_{i}K(x_{i},x_{j})$$
(3) Constructive decision function:
  (12) $$s(x)=sign(\sum_{i=1}^{N}\alpha_{i}^{*}y_{i}K(x,x_{i})+b^{*})$$

Step: 2. Because the integration of multiple support vector machines is better than that of a single support vector machine, the two models with the best SVM classification accuracy under different feature extraction are selected to predict the unlabeled image. If the two models form a consistent prediction for the same unlabeled image, the sample is selected as the quasi-label sample to increase the capacity of the neural network training set.

(13) $${\tilde{y}}_{l+u}=g(s({\tilde{x}}_{l+u}))$$

Step: 3. The labeled sample data set and quasi-labeled sample data set are combined to form an enhanced training set ATS.

(14) $$ATS=D_{T}^{S}\cup D_{u}$$

Step: 4. The augmented training set is used to train and solve the selected deep convolutional neural network classification model. This process realizes knowledge transfer from sparse labeled sample sets to deep neural network classifiers.

### 3.2. Deep Neural Network Classifier Model Construction

The main objective of this section is to select the appropriate neural network and build the model and solve the parameters. The selection of suitable neural network has a great impact on the results of image classification. The selected neural network is constructed and trained based on a large number of label samples obtained by knowledge transfer of a support vector machine.

**Figure 5:**
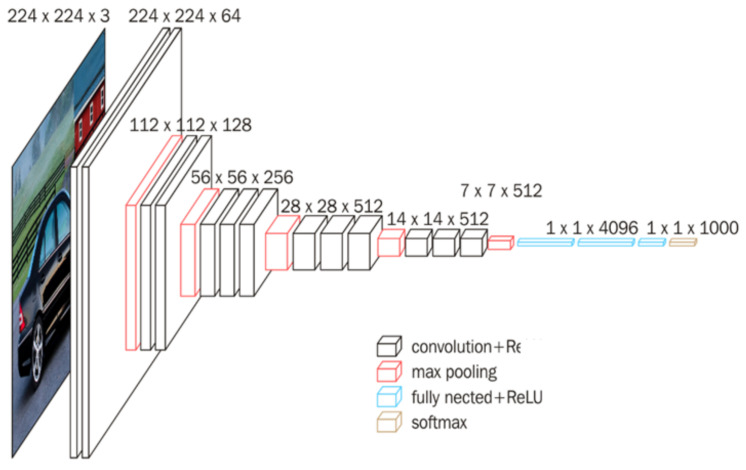
VGG16 network structure.

Vgg16 is a neural network that focuses on building convolution layers. The number 16 means that the network contains 16 convolution layers and full connection layers. Vgg16 has a regular network structure, with several convolution layers followed by a pool layer that can compress the size of the image. The pool layer reduces the height and width of the image. At the same time, there is a certain law in the change in the number of filters in the convolution layer. Because vgg16 shows good performance in various image classification, this network is selected as the basic model of this paper [27,28]. The network structure diagram of vgg16 is shown in Figure 5.

The neural network classification task has *n* training samples to be assumed, and the training set samples are denoted as $\left\{\left(x^{\left(1\right)},y^{\left(1\right)}\right),\left(x^{\left(2\right)},y^{\left(2\right)}\right),\cdots ,\left(x^{\left(n\right)},y^{\left(n\right)}\right)\right\}$, where $x^{n}$ represents the nth training sample, $y^{\left(n\right)}$ represents the true label of the sample, $y^{\left(n\right)}\in \left\{1,2,\ldots ,c\right\}$. c represents the number of classification task categories. For a given sample x, p(y=j|x) represents the probability of sample x in each class of classification results. Use the function $h_{\theta }(x)$ to represent p(y=j|x):

(15) $$h_{\theta }(x^{\left(i\right)})=\left[\begin{array}{c} p(y^{\left(i\right)}=1|x^{\left(i\right)};\theta )\\ p(y^{\left(i\right)}=2|x^{\left(i\right)};\theta )\\ \vdots \\ p(y^{\left(i\right)}=C|x^{\left(i\right)};\theta ) \end{array}\right]=\frac{1}{\sum_{j=1}^{c}e^{\theta_{j}x^{\left(i\right)}}}\left[\begin{array}{c} e^{\theta_{1}x^{\left(i\right)}}\\ e^{\theta_{2}x^{\left(i\right)}}\\ \vdots \\ e^{\theta_{c}x^{\left(i\right)}} \end{array}\right]$$

where $\theta_{1},\theta_{2},\cdots ,\theta_{c}$ represents the parameters of the model, and the specific form of θ is as follows:

(16) $$\theta =\left[\begin{array}{c} \theta_{1}\\ \theta_{2}\\ \vdots \\ \theta_{c} \end{array}\right]$$

At this point, the cost function of the classifier is:

(17) $$J\left(\theta \right)=-\frac{1}{n}\left[\sum_{i=1}^{n}\sum_{j=1}^{c}1\left\{y^{\left(i\right)}=j\right\}\log\frac{e^{\theta_{j}x^{\left(i\right)}}}{\sum_{l=1}^{c}e^{\theta_{l}x^{\left(i\right)}}}\right]$$

where $1\left\{\cdot \right\}$ is the indicative function, $1\left\{true\right\}=1,1\left\{false\right\}=0$.

## 4. Digital Simulation Example and Algorithm Performance Analysis

This section mainly introduces the data sets and experimental environment used in the experiment, and describes in detail the analysis and influence of different feature extraction methods on the accuracy of SVM image classification, the influence and analysis of the number of quasi label data sets on the accuracy of neural network classification, and the analysis and comparison of the accuracy of different models in the case of small samples.

### 4.1. Experimental Preparation

In order to verify the effectiveness of the algorithm proposed in this paper, the caltech256 data set collected by California Institute of technology is used. In this dataset, pictures are divided into 256 categories, and the pictures in each category range from 80 to 800. Six kinds of images are selected for experiments, namely, aircraft, face, horse, ladder, motorbike, and T-shirt. The quantity of each type of image data and its labels are shown in Table 1:

Due to the unbalanced number of image samples per class, when generating pseudo-labels, the network tends to skew toward classes with a larger number of training sets. In order to prevent this from happening, two fixed numbers of samples were randomly selected from each type of image samples as labeled samples, and the labeled sample sizes of each type of image are 24 and 36, respectively. The labeled samples were divided into a training set and validation set with a ratio of 2:1, and then a threefold cross-validation experiment was performed to train the support vector machine. This means that the number of training sets is 16 when the labeled sample capacity is 24, and the number of training sets is 24 when the labeled sample capacity is 36.

### 4.2. Analysis and Influence of Different Feature Extraction Methods on SVM Image Classification Accuracy

**Figure 6:**
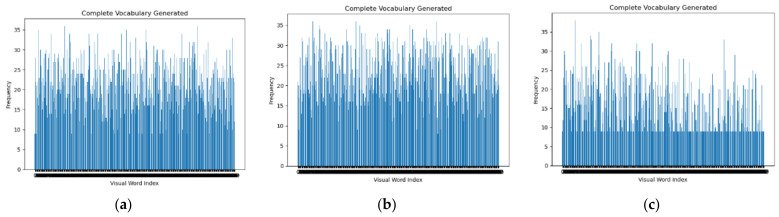
(**a**) Feature extraction histogram of SIFT; (**b**) Feature extraction histogram of Hog; (**c**) Feature extraction histogram of Canny.

Since different feature extraction methods and some important parameters have a great impact on the SVM classification performance, a large number of experimental analyses were to select SVMs with high classification accuracy. Figure 6 visualizes the frequency histograms of image visual words under different image feature descriptor underlying feature extraction methods. The visual word frequency histogram was extracted under the condition of k = 400.

**Figure 7:**
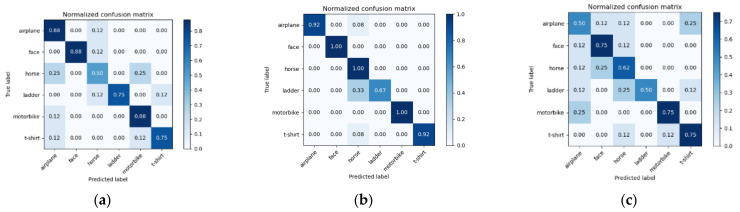
(**a**) Confusion matrix of SIFT_BOVW_SVM; (**b**) Confusion matrix of Hog_BOVW_SVM; (**c**) Confusion matrix of Canny_BOVW_SVM.

From the visual feature extraction histogram, it can be seen that the visual word histogram frequency of sift and hog feature extraction is higher than canny frequency, so sift and hog can better reflect the characteristics of the image. Figure 7 shows the validation results of SVM trained under different feature extraction and k = 400 when the sample size of each type of labeled image is 24 and divided into training set and validation set in a ratio of 2:1 for cross validation. The confusion matrix was used to represent and visualize them.

**Figure 8:**
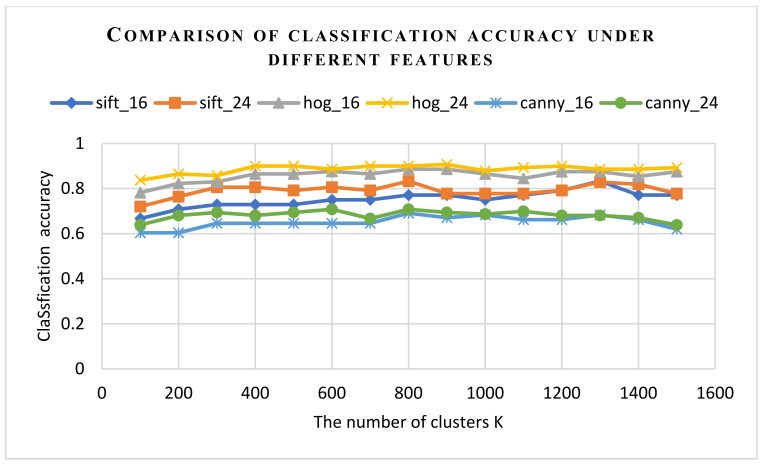
Comparison of classification accuracy under different features.

In order to find the most suitable parameter value k, this paper conducted a lot of experiments in different feature extraction situations. In order to ensure the reliability of the experimental data, the data sets with labeled sample sizes of 24 and 36 were randomly divided into three parts, two of which were used as training sets and one was used as test sets. The average classification accuracy obtained by the threefold crossover experiment was used as the basis to measure the classification performance of the support vector machine: 16 in the table represents the number of training sets when the labeled sample size is 24, and 24 in the table represents the number of training sets when the labeled sample size is 36. Table 2 gives the specific overall classification results. Figure 8 visualizes the data in Table 2 with a line chart.

When a CNN was trained on a dataset with noisy labels, the neural network will overfit to the noisy data because the deep network can learn and memorize any training dataset. When selecting a prediction model to label unlabeled samples, two SVM classifiers with good classification performance were selected as the labelers. At the same time, two SVM classifiers were used to label the same sample. If the prediction results were the same, the label was used as the quasi-label of the unlabeled sample.

In order to make the correct rate of pseudo-labels predicted by SVM at a high level, different k values were selected when making the bag-of-words model. The size of the visual dictionary was determined by the number of clusters k. A suitable value of k makes the extracted feature vector more representative of image features. If the dimension of the visual dictionary is too small, the difference between different types of images cannot be clearly represented. If the dimension is too large, it will lead to the redundancy of features, and the key information of the image will be too scattered, which will cause the disaster of dimensionality and seriously affect the classification efficiency of the image. The experimental results show that when k = 100 to 400, the classification accuracy of the support vector machine SVM under the bag-of-words model of HOG is continuously improved. At k = 400 to 1500, the SVM image classification accuracy tends to be stable, while the feature extraction time and model training time continue to increase. It can be seen from Figure 8 and Table 2 that the image classification accuracy under this feature can reach up to 91%. In the case of small samples, the texture and contour information extracted by HOG can be used to effectively represent image features, so that support vector machines can express good classification performance. Compared with the image features of SIFT and Canny, the dimension of the image features extracted by HOG is small, and the model training time is also less. In general, the SVM pseudo-labels trained under the HOG feature had the highest accuracy and efficiency. When k = 100 to 800, the classification accuracy of support vector machine SVM under the bag-of-words model of SIFT continuously improved. When k = 800 to 1500, with the increase in the k value, the model training time increased continuously, but the classification effect is not significantly enhanced. In this case, the image classification effect is not proportional to the k value. Compared with the HOG and SIFT feature extraction methods, the classification accuracy of the support vector machine trained by the features extracted by Canny reaches the optimum and tends to be stable with the increase in the k value when k = 800; after which, the classification accuracy of SVM will not improve significantly with the increase in the k value. Compared with the classification accuracy under HOG and SIFT features, the classification level under this feature is lower. Therefore, the support vector machine model under this feature extraction was not used as the prediction model. In order to verify the influence of different numbers of small samples on the classifier, this paper selected 16 and 24 training sets to train the support vector machine model. The experimental results show that the support vector machine model trained with the number of training sets of 24 is better than the support vector machine model with the number of 16.

### 4.3. Influence of the Number of Quasi-Label Data Sets on the Classification Accuracy of Neural Network

The support vector machine model with the highest accuracy to label and screen unlabeled image samples was used. The training set of quasi-labeled image samples and labeled image samples was fed into a fine-tuned convolutional neural network VGG16 for training. In the experiments, five augmented training sets with different capacities were constructed. The number of boosted training sets increased by 500 each time. The augmented training set was used to train the convolutional neural network. The remaining one-third of the labeled image samples in each category were used as the validation set of the convolutional neural network, and the validation set was used to test the effectiveness of the small-sample image classification algorithm based on the convolutional neural network and support vector machine proposed in this paper. The images need to be preprocessed before the neural network can be trained. First, the size of the image was cropped to 224 × 224. Then, the cropped image were flipped horizontally with probability *p* = 0.5 and normalized. VGG16 uses the Adam optimization algorithm during training. VGG16 uses iterative training, the maximum number of iterations is 50,000 epochs, and the learning rate is set to 0.0001. The results are shown in the following Table 3:

It can be seen from the table that when the number of labeled samples is 16 and 24 to increase the number of quasi-labeled data sets, the classification accuracy of the same neural network on the validation set is not much different. The reason for this is that the total number of training sets is not much different. However, the enlargement of the training set has a great impact on the training of the neural network. With the increase in the number of quasi-labeled data, the accuracy of the neural network on the validation set continues to increase, indicating that the knowledge learned by the support vector machine under the small sample data was successful. Migrating to a convolutional neural network improved the over-fitting phenomenon caused by low classification accuracy of neural network under small label samples.

### 4.4. Comparison of Classification Accuracy of Different Models in the Case of Small Samples

**Figure 9:**
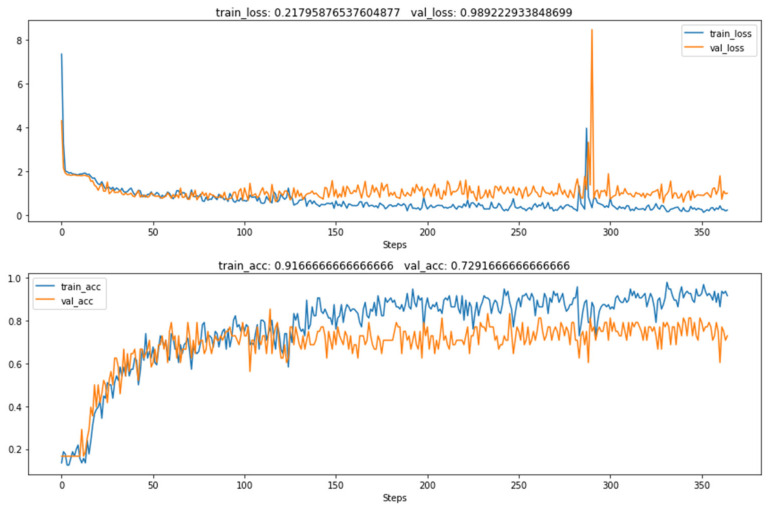
Vgg16 network training process under small samples.

The highest average classification accuracy on the validation set of the SVM trained under three different feature descriptors, the VGG16 network under parameter initialization and the knowledge transfer method proposed in this paper are compared in this section. The comparison is shown in Table 4. Figure 9 shows the change in the loss function and the accuracy rate during the VGG16 training process using only small samples.

It can be seen from the table and figure that the VGG16 directly trained with small samples has serious overfitting. The accuracy of the training set is 91.66% and the accuracy of the test set is 72.91%. Due to the simple feature extraction method of traditional image classification methods, the classification accuracy is between 70% and 90%. The convolutional neural network image classification effect based on knowledge transfer is better, and the classification accuracy can reach 93.4%. It is 3% higher than the highest classification accuracy of the traditional machine learning classifier Support Vector Machine (SVM). Compared with the deep convolutional neural network in the small sample case, the VGG16 model trained with the quasi-labeled sample dataset can learn better discriminative features and improve the classification accuracy by 20%. This means that the knowledge learned from the artificial bag-of-words model and the shallow model SVM can be successfully transferred to the pretrained convolutional neural network in the form of predicted sample labels. The effectiveness of this method in solving the problem of low image classification accuracy in the case of small samples is verified in the experiments.

## 5. Summary and Prospects

This paper mainly introduces a new method of deep convolutional neural network image classification based on knowledge transfer in the environment of small label samples. This method mainly combines different feature extraction methods with different advantages, and integrates multiple traditional image classification methods to predict unlabeled images. The number and accuracy of quasi-labeled samples increased. The augmented training set enables efficient training of convolutional neural networks. The knowledge learned by the shallow model support vector machine was successfully transferred to the convolutional neural network model, which improved the robustness and generalization ability of the convolutional neural network model. Experimental verification shows that the proposed method has better classification performance than traditional image classification methods. Our method is 3% higher than the highest average classification accuracy of HOG_BOVW_SVM. Compared with the convolutional neural network with parameter initialization under the few-label samples, the average classification accuracy is 20% higher. In the future research, since different classifiers have different characteristics, if the classification performance can be better by combining multiple classifiers, the neural network will learn better knowledge. In addition, the feature extraction information of the trained neural network can be used to embed multiple classifiers into the neural network for model fusion to further improve the accuracy of image classification. As the structure of the neural network gets deeper and deeper, the gradient descent algorithm can be replaced by the Kalman filter for adaptive updating [29]. This is a direction that can be considered in the future.

## Figures and Tables

**Table 1:** Types, quantities, and labels of images.

Image Type	Quantity	Label
Airplane	800	1
Face	435	2
Horse	270	3
Ladder	242	4
Motorbike	798	5
T-shirt	358	6

**Table 2:** Classification accuracy of SVM verification set under different characteristics.

	Model	Sift		Hog		Canny	
Parameter							
Number of cluster	kernel function	16	24	16	24	16	24
100	linear	0.667	0.72	0.782	0.837	0.604	0.639
200	linear	0.708	0.764	0.823	0.865	0.604	0.681
300	linear	0.729	0.806	0.83	0.858	0.646	0.694
400	linear	0.729	0.806	0.865	0.90	0.646	0.681
500	linear	0.729	0.792	0.865	0.90	0.646	0.694
600	linear	0.75	0.806	0.876	0.886	0.646	0.708
700	linear	0.75	0.792	0.865	0.90	0.646	0.667
800	linear	0.771	0.833	0.886	0.90	0.691	0.708
900	linear	0.771	0.778	0.886	0.907	0.671	0.694
1000	linear	0.75	0.778	0.865	0.879	0.683	0.687
1100	linear	0.771	0.778	0.845	0.893	0.662	0.699
1200	linear	0.792	0.792	0.875	0.90	0.662	0.681
1300	linear	0.833	0.829	0.875	0.886	0.683	0.681
1400	linear	0.771	0.819	0.854	0.886	0.662	0.671
1500	linear	0.771	0.778	0.875	0.893	0.62	0.639

**Table 3:** Classification accuracy of vgg16 verification set under different capacity enhancement training sets.

Number of Training Set		
Number of Labeled Samples	Quasi Label Capacity Increase Quantity	Val_acc
16/24	0	72.9%/73.5%
16/24	500	74.5%/74.6%
16/24	1000	78.5%/79.3%
16/24	1500	83.5%/83.7%
16/24	2000	89.6%/88.1%
16/24	2500	92.5%/93.4%

**Table 4:** Classification accuracy under small label samples of different models.

Modle	Training Set Sample Size	
	16	24
SIFT_BOVW_SVM	83.3%	83.3%
HOG_BOVW_SVM	87.5%	90.7%
Canny_BOVW_SVM	69.1%	70.8%
VGG16	72.9%	73.5%
BOVW_SVM_VGG16	92.5%	93.4%
